# The influence of corpulence on the mechanical properties of the human heel fat pad in elderly

**DOI:** 10.1186/1757-1146-5-S1-P20

**Published:** 2012-04-10

**Authors:** Frank Lindner, Günther Schlee, Thomas L Milani

**Affiliations:** 1 Institute of Sport Science, Human Locomotion, Chemnitz University of Technology, Chemnitz, Free State of Saxony, 09107, Germany

## Background

The human heel fat pad (HFP) is an effective shock absorber [1]. A remodelling of body fat begins from the fifth decade of the human life. Diet may negatively influence this distribution and consequently lead to corpulence in elderly persons. Alcantara and colleges reported that fat content in the HFP increases with obesity [2]. Hence, the purpose of this investigation was to determine the effect of corpulence on the mechanical properties of the HFP in elderly. We hypothesized, that corpulence alters mechanical properties of the HFP compared to normal weighted elderly persons.

## Materials and methods

**Figure 1:**
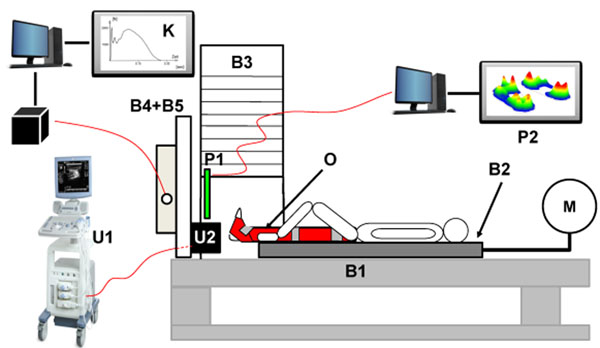
shows the loading device (measurement accuracy of the system is 0.09 mm and 18 kPa). Details on design of the loading device: **B1** foundation, **B2** carriage, **B3** tower of weights, **B4+B5** separated footrest with integrated GRF-platform (1000 Hz) to measure Vertical GRF, **M** microprocessor controlled step motor for the velocity controller of the carriage, **O** leg brace; **P1+P2** emed pedography platform (resolution 2 sensors/cm², frequency 50 Hz), **U1** Ultrasound device (axial resolution 0.07 mm), **U2** fixture with integrated ultrasound transducer

Twenty-three healthy elderly corpulent (age 61 ± 6 yrs, height 171 ± 8 cm, BMI 28 ± 2, weight 82 ± 7 kg) and nineteen non-corpulent men and women (age 61 ± 7 yrs, height 168 ± 8 cm, BMI 23 ± 1, weight 65 ± 7 kg) took part in the experiment. A loading device was used for in vivo testing of the HFP (Figure 1). Parameters were measured under two different impact velocities (low 2 mm/s, fast 10 mm/s). Several mechanical variables (unloaded and loaded HFP thickness, stiffness S, elasticity ε) were calculated.

## Results

Thickness variables of corpulent subjects were significantly higher compared to the non-corpulent. The stiffness was found to have a nonlinear behaviour in which corpulent subjects show lower stiffness in the final stage. There was no significant difference in ε.

## Conclusions

Increased HFP thickness is an adaption process to increased body weight, suggesting that an accumulation of fat cells with good blood supply at micro chamber structure may have occurred. This process protects the macro chamber structure of the HFP against overload that is transferred by micro chambers. Therefore, the micro chamber structure of corpulent elderly may be mechanically more sensitive and damageable than in non-corpulent subjects.
